# Postoperative ileus after emergency surgery for acute bowel obstruction: a case-control study of predictors and impact on recovery

**DOI:** 10.1007/s00423-025-03851-0

**Published:** 2025-09-01

**Authors:** Anca-Laura Amati, Niklas Kümmel, Nicoleta Negruta, Martin Reichert, Thilo Schwandner, Julia Noll, Jaqueline Braun, Veronika Grau, Sebastian Petzoldt, Andreas Hecker

**Affiliations:** 1 Department of General, Visceral, Thoracic and Transplant Surgery, University Hospital of Giessen, Justus-Liebig University Giessen, Rudolf-Buchheim-Strasse 7, 35392 Giessen, Germany; 2 Department of General and Visceral Surgery, Asklepios Clinic Lich, Goethestrasse 4, 35423 Lich, Germany; 3 Department of Anesthesiology, Intensive Care Medicine and Pain Therapy, University Hospital of Giessen, Justus-Liebig University Giessen, Rudolf-Buchheim-Strasse 7, 35392 Giessen, Germany

**Keywords:** Acute bowel obstruction, Postoperative ileus, Emergency surgery, Predictors, Postoperative complications

## Abstract

**Purpose:**

Acute bowel obstruction (ABO) is a common indication for emergency abdominal surgery, often performed on a severely dilated intestine with compromised barrier function. The recovery of bowel motility in this acute setting differs from elective surgery and remains insufficiently investigated. Yet, its understanding is crucial for improving perioperative care in surgical emergencies. This study aimed to identify risk factors for postoperative ileus (POI) after emergency surgery for ABO and to explore its impact on postoperative outcomes.

**Methods:**

We retrospectively analyzed 466 patients who underwent emergency surgery for ABO between 2014 and 2020, of whom 156 developed POI. POI was defined as the absence of postoperative bowel movement requiring intravenous prokinetic therapy following an insufficient response to initial conservative measures, including oral laxatives. Perioperative predictors for POI were identified by univariate and multivariate logistic regression. The association between POI and adverse postoperative outcomes was examined using correlation analysis.

**Results:**

Multivariate analysis revealed male sex (*p* = 0.0009), chronic steroid therapy (*p* = 0.0064), high postoperative SOFA score (*p* = 0.0037), and elevated CRP levels on postoperative day two (*p* = 0.048) as independent predictors for POI. Patients with POI had significantly higher rates of postoperative respiratory (*p* < 0.0001) and surgical complications (*p* = 0.0014).

**Conclusion:**

Our findings suggest sex-dependent differences and an involvement of inflammatory processes in bowel function recovery following emergency surgery for ABO. POI in this setting is associated with increased risk of postoperative respiratory complications and surgical morbidity. These results highlight the need for targeted preventive strategies and form a solid foundation for future prospective studies aimed at optimizing perioperative care and reducing POI-related morbidity in surgical emergencies.

**Supplementary Information:**

The online version contains supplementary material available at 10.1007/s00423-025-03851-0.

## Introduction

Acute bowel obstruction (ABO) is the most frequent surgical condition leading to emergency abdominal surgery [1, 2]. It encompasses a variety of intra-abdominal pathologies, ranging from adhesions to incarcerated hernias, united by the shared feature of a mechanically occluded intestinal lumen. This obstruction causes progressive dilation of the upstream bowel, leading to edema, congestion, and increased intestinal wall permeability [3]. When surgical manipulation is added to an already compromised bowel, delayed postoperative recovery becomes highly likely. Indeed, impaired return of bowel motility is a near pathognomonic consequence of major abdominal surgery [4, 5]. Its full-scale clinical manifestation - postoperative ileus (POI) - represents the functional counterpart of ABO within the ileus spectrum of disease. Even in elective procedures involving a physiologically normal bowel, POI occurs in 10–25% of cases [5–8]. It significantly contributes to patient discomfort through abdominal distension and vomiting, prolongs hospitalization, and increases postoperative morbidity and mortality [7, 9].

As opposed to the elective procedures, emergency surgery for ABO is performed on a pathologically altered intestine [3]. The degree in which this affects the postoperative recovery of bowel function remains largely unexplored, as most data concerning POI derive from elective surgery. The extensive research on POI following elective surgery reflects the overall clinical and financial burden of the condition and has led to an improved mechanistic understanding of the processes enabling POI, while simultaneously identifying possible therapeutic targets [5, 6, 8, 10]. A major contributor to POI is intestinal inflammation triggered by the surgical manipulation of the bowel [5, 6, 11]. Interestingly, the degree of manipulation correlates with the extent of postoperative bowel inflammation and with the frequency and severity of POI [12–14]. These findings align with the clinical observation showing a decreased risk for POI after laparoscopic surgery [15, 16]. Although even high-risk surgical patients can benefit from minimally invasive techniques [16], a recent survey of the World Society of Emergency Surgery showed that laparoscopy remains underutilized in the emergency setting, often despite available technical expertise [17].

Moreover, not all patients are suitable candidates for laparoscopy. The risk of iatrogenic bowel injury is higher in individuals with previous laparotomies or pelvic radiotherapy [18–21]. Therefore, minimizing bowel handling is not always feasible. This highlights the need to investigate additional factors that contribute to POI following emergency surgery for ABO, to help identify patients at elevated risk of postoperative complications. Such patients may benefit from targeted pharmacological interventions, such as peripheral µ-opioid receptor antagonists (e.g., alvimopan) or selective 5-HT4 receptor agonists (e.g. prucalopride, cisapride) [7, 8].

As efforts to implement enhanced recovery and quality improvement programs for emergency surgery gain momentum, it is crucial that the recommendations within these programs are derived specifically from emergency surgery patients, rather than being extrapolated from elective surgery. In recognition of data scarcity on this particular issue, we conducted the current study with the aim to identify potential risk factors for delayed bowel function recovery and assess the clinical implications of POI in patients undergoing emergency surgery for ABO. Given the retrospective nature of the analysis, our findings should be interpreted as hypothesis-generating and warrant validation in future prospective studies.

## Materials and methods

All consecutive patients (≥ 18 years of age) undergoing emergency surgery for ABO between January 2014 and December 2020 in the Department of General, Visceral, Thoracic and Transplant Surgery of the University Hospital of Giessen were retrospectively evaluated. The study was registered with the German Clinical Trials Register (https://drks.de/search/en/trial/DRKS00034851) and the manuscript written in accordance with the STROBE guidelines [22].

### Inclusion and exclusion criteria

The primary inclusion criterion was an intraoperatively confirmed, complete mechanical obstruction of the bowel lumen, along with available postoperative documentation of bowel function, including interventions aimed at stimulating bowel peristalsis. Given the lack of a universally accepted definition of POI [23], we defined it as the necessity for intravenous administration of prokinetic agents to stimulate bowel motility following an insufficient response to initial conservative measures, including oral laxatives. This definition is consistent with previously published criteria [24].

**Fig. 1:**
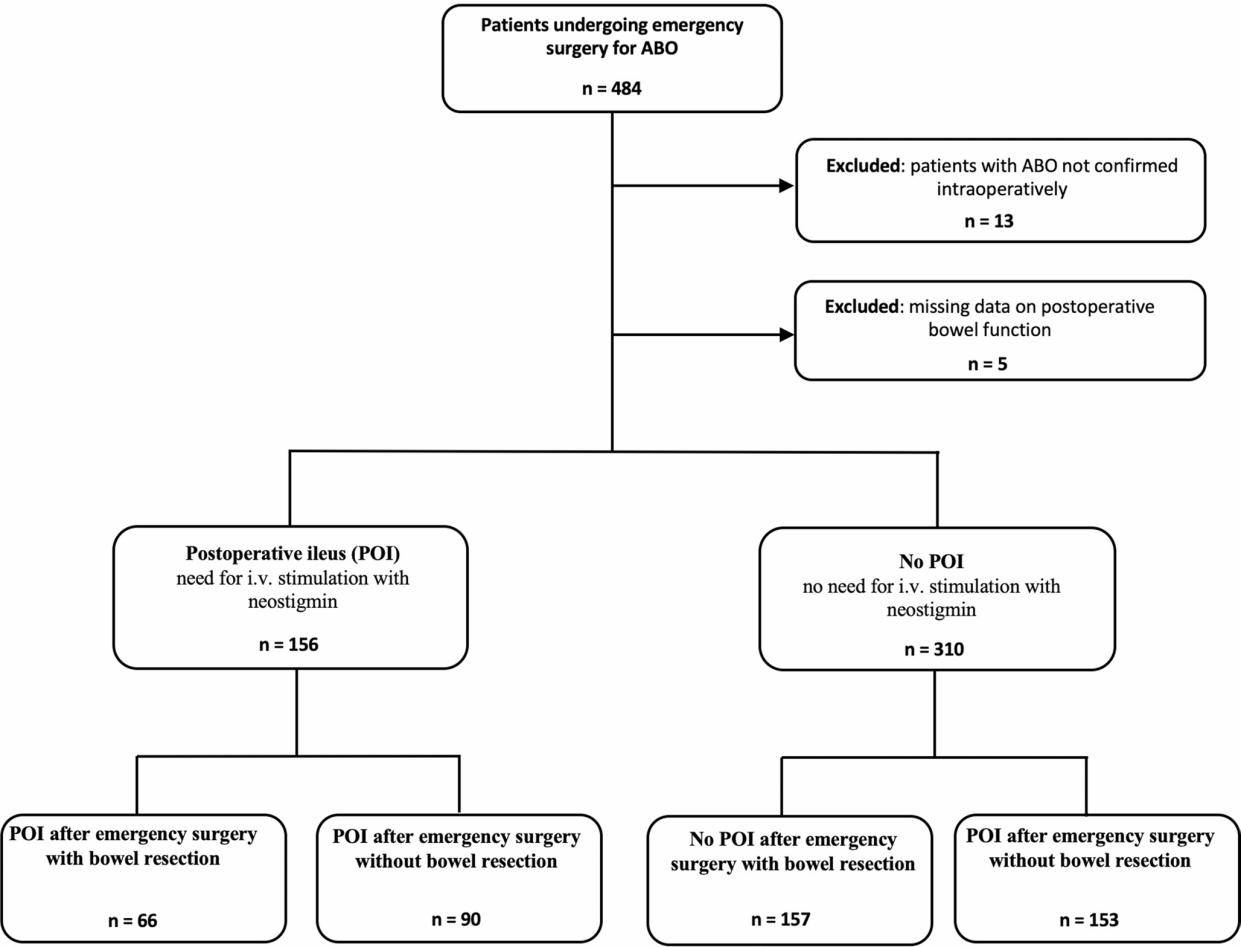
Patient selection and subgroup definition. 484 patients undergoing emergency surgery for acute bowel obstruction (ABO) were screened for study inclusion. 18 patients were excluded due to false preoperative diagnosis or lack of data concerning postoperative bowel function. The remaining 466 patients were divided into two subgroups, based on whether postoperative ileus (POI) requiring therapeutic intervention ensued (156 patients) or not (310 patients). The two patient subgroups were further divided depending on whether the emergency surgical procedure involved bowel resection or not

Patients were excluded if intraoperative findings refuted the clinical or radiological suspicion of ABO, or if they presented with incomplete obstruction managed electively by adhesiolysis. Further exclusion criteria included the absence of postoperative bowel function data and the use of non-surgical interventions - such as endoscopic stenting or percutaneous gastrostomy - performed for palliative decompression. The selection process is illustrated in Fig. 1.

### Study variables

Preoperative data included patient demographics, pre-existing medical conditions with Charlson Comorbidity Index (CCI) calculation, American Society of Anesthesiologists (ASA) fitness grade, history of abdominal surgery, and the use of anticoagulant or immunosuppressive medication that could affect postoperative morbidity.

Preoperative laboratory parameters assessed inflammation, liver, and kidney function as part of the standard blood analysis for surgical emergencies. Intraoperative data included the causes of bowel obstruction, surgical approach, performed surgical procedures, surgery duration, and blood loss. Postoperative assessment included the Sequential Organ Failure Assessment (SOFA) score within the first 24 h after surgery and C-reactive protein (CRP) levels from postoperative day (POD) 1 to day 5.

### Outcome measures

The primary outcome parameter was the occurrence of POI, defined as the requirement for intravenous prokinetic therapy. Based on this criterion, the patients were categorized into a POI group and a no POI group.

Our hospital’s standard treatment for altered bowel motility begins with laxatives and progresses to gastrografin, administered orally or via a nasogastric tube (NGT). The final escalation step, that defines POI in this study, consists of administering intravenous metoclopramide and neostigmine if bowel function does not improve. The acetylcholinesterase inhibitor neostigmine is well-studied in the context of POI following abdominal surgery [25]. In addition to pharmacologic stimulation, early mobilization and oral intake were encouraged within the constraints of the patients’ clinical condition. Other medications known to influence bowel motility, such as the µ-opioid receptor antagonist alvimopan, were not used in our patients.

Secondary outcome measures were duration of ICU stay, mortality and postoperative morbidity, either surgical, stratified according to the Clavien-Dindo classification [26] or medical with a focus on respiratory complications (postoperative pneumonia, need for non-invasive ventilation after initial extubation, need for reintubation). Also analyzed were the need of supplementing caloric intake through parenteral nutrition, the delayed removal of the NGT after POD3, the readmission rate to the ICU, and the rate of successful release to the initial home environment.

## Statistical analyses

Data analysis was performed using GraphPad Prism (Version 9 for Windows, GraphPad Software, San Diego, CA, USA, www.graphpad.com). Continuous variables are presented as median and interquartile range (IQR) and were analyzed using the Mann-Whitney U test. Categorical variables are shown as numbers with percentages, n (%), and were compared using a chi-squared test or Fisher’s exact test, as appropriate. Associations between preoperative as well as intraoperative parameters and POI were investigated by univariate logistic regression. Variables with statistically significant association on univariate analysis were included in a multivariable logistic regression model. To address multicollinearity, we applied a conservative threshold of 2.5 for the calculated variance inflation factors (VIF) [27].

To analyze the postoperative development of laboratory parameters over time in patient subgroups we performed a two-way repeated measures (mixed model) ANOVA, applying the Bonferroni multiple-comparison correction. Relationships between POI and postoperative complications were tested by correlation analysis, reporting Spearman’s correlation coefficient (r) as well as the according p values. P values of ≤ 0.05 (two-sided) were considered statistically significant.

## Results

1. **Study outline and subgroup definition**.

During the study period, 484 patients underwent emergency surgery with the indication of ABO. 13 patients were excluded for not having the diagnosis of a mechanical bowel obstruction confirmed intraoperatively. Another 5 patients were excluded due to lack of data on postoperative bowel function (Fig. 1).

Based on whether postoperative neostigmine administration was required, the remaining 466 patients were divided into two subgroups, with 156 patients developing POI and 310 patients not experiencing this complication.

For analyzing the impact of the type of surgical procedure on bowel recovery, the two already defined patient subgroups were further sub-divided into patients receiving bowel resection during emergency surgery and patients for whom the obstruction was resolved without bowel resection, namely through adhesiolysis, detorsion or reposition of hernia content (Fig. 1).

2. **Pre- and intraoperative characteristics of patients undergoing emergency surgery for ABO**.

Analysis of patient demographics showed a significantly higher percentage of male patients developing POI (69.2% vs. 49%). Patients with POI also had a significantly higher BMI. There were no significant differences regarding comorbidities or risk-scores reflecting comorbidities between the two patient subgroups. A similarly high percentage of patients in both subgroups had previous abdominal surgery (83.9% vs. 76.4%). Chronic steroid intake was significantly more frequent in patients with POI. These patients also had significantly higher CRP and creatinine levels but lower albumin levels than patients not developing POI (Table 1).

**Table 1:** Baseline characteristics of patients undergoing emergency surgery for acute bowel obstruction

Patient characteristics	POI (*n* = 156)	No POI (*n* = 310)	*p* value
Demographics			
Age, years (IQR)	71 (57–78)	67 (53.7–77)	0.1231
Male sex, n (%)	108 (69.2%)	152 (49%)	**< 0.0001**
BMI, kg/m^2^ (IQR)	26 (22.3–29.4)	24.8 (21.4–28)	**0.0417**
Underlying conditions			
Chronic pulmonary disease, n (%)	39 (25%)	55 (17.7%)	0.0679
Malignancy, n (%)	59 (37.8%)	131 (42.2%)	0.3704
Chronic liver disease, n (%)	18 (11.5%)	30 (9.6%)	0.5229
Chronic kidney disease, n (%)	38 (24.3%)	55 (17.7%)	0.1101
Cardio-vascular disease, n (%)	107 (68.59%)	192 (61.9%)	0.1833
Diabetes, n (%)	33 (21.15%)	47 (15.1%)	0.1186
Chronic inflammatory disease, n (%)	16 (10.2%)	31 (10%)	0.9309
CCI, % (IQR)	21% (0% − 77%)	53% (0% – 90%)	0.3981
Previous abdominal surgery, n (%)	131 (83.9%)	237 (76.4%)	0.0600
Number of previous abdominal surgeries, (IQR)	2 (1–3)	2 (1–3)	0.9270
ASA-score, (IQR)	3 (3–3)	3 (2–3)	0.9209
Previous medication			
Therapeutic anticoagulation, n (%)	27 (17.3%)	40 (12.9%)	0.2100
Platelet aggregation inhibitors, n (%)	41 (26.2%)	79 (25.4%)	0.9107
Chronic steroid therapy, n (%)	21 (13.4%)	15 (4.8%)	**0.0016**
Immunosuppressants, n (%)	11 (7%)	12 (3.8%)	0.1728
Preoperative laboratory parameters			
Leukocyte count, 10^9^/L (IQR)	8.5 (5.7–13.5)	9.9 (6.7–14.2)	0.1020
Hb, g/L (IQR)	122 (107–139)	124 (106–139)	0.7490
CRP, mg/L (IQR)	53.5 (13.6–137)	31.3 (10.9–90.5)	**0.0049**
Albumin, g/L (IQR)	33.40 (28.3–38.3)	35,00 (29. – 40.9)	**0.0213**
Creatinine, mg/dL (IQR)	1 (0.8–1.5)	0.9 (0.7–1.3)	**0.0056**
Bilirubin, mg/dL (IQR)	0.8 (0.4–1.1)	0.7 (0.4–1)	0.1758

Bold text values represent p values less than 0.05

Abbreviations: *POI* postoperative ileus, *IQR* interquartile range, *BMI* body mass index, *CCI* Charlson comorbidity index, *ASA* American Society of Anesthesiologists, *Hb* hemoglobin, *CRP* C-reactive protein.

There were significant differences concerning the intraabdominal pathologies leading to bowel obstruction. Patients with POI had a higher occurrence of multiple interenteric adhesions (42.9% vs. 27.1%), while malignant obstruction was more frequent in patients without POI. The surgical treatment of the bowel obstruction implied bowel resection in 42.3% of the patients with POI and 50.6% of the patients without POI, with small, large or combined intestinal resections distributed in similar proportions within the two patient subgroups. The most prevalent surgical approach was median laparotomy. A laparoscopic resolution of ABO was achieved in only 1.9% of patients in the POI group and 1% in the no POI group. Conversion from an initially laparoscopic approach to laparotomy was required in 1 patient in the POI group and 7 patients in the no POI group. Given the overall low rates of laparoscopy in both groups, no significant differences were observed with respect to the surgical approach (Table 2).

**Table 2:** Intraoperative and early postoperative characteristics of patients undergoing emergency surgery for acute bowel obstruction

Intra- and early postoperative characteristics	POI (*n* = 156)	No POI (*n* = 310)	*p* value
Intraoperative characteristics			
Cause of obstruction			
Primary tumor, n (%)	4 (2.6%)	29 (9.4%)	**< 0.0001**
Peritoneal metastasis, n (%)	3 (1.9%)	31 (10%)	
Intestinal inflammation, n (%)	8 (5.1%)	4 (1.3%)	
Single strand adhesions, n (%)	44 (28.2%)	78 (25.2%)	
Multiple adhesions, n (%)	67 (42.9%)	84 (27.1%)	
Hernia, n (%)	11 (7.1%)	56 (18.1%)	
Other, n (%)	19 (12.2%)	28 (9%)	
Surgical procedure			
Surgical approach			
Laparotomy, n (%)	152 (97.4%)	300 (96.8%)	0.3216
Laparoscopy, n (%)	3 (1.9%)	3 (1%)	
Conversion from laparoscopy to laparotomy	1 (0.6%)	7 (2.3%)	
No bowel resection, n (%)	90 (57.7%)	153 (49.4%)	0.0891
Bowel resection, n (%)	66 (42.3%)	157 (50.6%)	
Small bowel resection, n (%)	37 (56.1%)	96 (61.6%)	0.5466
Large bowel resection, n (%)	16 (24.2%)	28 (17.8%)	
Both small and large bowel resection, n (%)	13 (19.7%)	33 (21.0)	
Intraoperative blood loss, mL (IQR)	170 (50–300)	100 (50–250)	0.2410
Operation time, min (IQR)	112 (74–174)	92.5 (65–147)	**0.0050**
Early postoperative characteristics			
ICU duration of stay > 24 h, n (%)	109 (69.8%)	177 (57.1%)	**0.0075**
Postoperative SOFA score, points (IQR)	2 (0–6)	1 (0–4)	**0.0003**
CRP POD 1, mg/L (IQR)	157.1 (93.8–223.2)	132.1 (70.9–180.8)	**0.0239**
CRP POD 2, mg/L (IQR)	226.7 (170.1–297.1)	189.5 (141.7–264.5)	**0.0004**
CRP POD 3, mg/L (IQR)	190.1 (136.8–253.1)	155.8 (103.3–216.5)	**0.0041**
CRP POD 5, mg/L (IQR)	117.9 (65.4–161.1)	88.34 (45.61–141.5)	**0.0043**

Bold text values represent p values less than 0.05

Abbreviations: *POI* postoperative ileus, *IQR* interquartile range, *ICU* intensive care unit, *SOFA* sequential organ failure assessment, *CRP* C-reactive protein, *POD* postoperative

Surgery took significantly longer in patients developing POI. Significant differences were also apparent in the early postoperative phase, with more frequently prolonged ICU stay (*p* = 0.0075), more severe organ dysfunction (*p* = 0.0003) and higher levels of systemic inflammation (*p* = 0.0004 for CRP on POD 2) registered in patients with POI (Table 2).

3. **Univariate and multivariate analysis of perioperative factors associated with POI after emergency surgery for bowel obstruction**.

The following variables showed a statistically significant association with POI in the univariate analysis (Table 3): male sex (*p* < 0.0001), chronic steroid therapy (0.0015), previous abdominal surgery (*p* = 0.0467), higher baseline CRP levels (*p* = 0.0145), lower baseline albumin (*p* = 0.0107), a longer duration of emergency surgery (*p* = 0.0048), a higher postoperative SOFA score (*p* = 0.0006), and higher CRP levels on POD 2 (*p* < 0.0001). Several other laboratory parameters (leukocyte count, hemoglobin, creatinine, and bilirubin) recorded between POD1 and POD 3 showed no significant association with POI (Supplementary Material, Table 1). As CRP levels on the first, second and third day after surgery are not independent variables, we chose to include the CRP levels on POD 2 into our analysis for reaching the highest level of significance in the univariate analysis (Supplementary Material, Table 1). In the multivariate analysis, male sex (*p* = 0.0009), chronic steroid therapy (*p* = 0.0064), a higher postoperative SOFA score (*p* = 0.0037), and higher CRP levels on POD 2 (*p* = 0.048) independently associated with POI (Table 3).

**Table 3:** Uni- and multivariable logistic regression analyzing perioperative risk factors for postoperative ileus after emergency surgery for bowel obstruction

Risk factor	Univariate		Multivariate	
	OR (95% CI)	*p*	OR (95% CI)	*p*
Age	1.009 (0.9964–1.021)	0.1671		
Sex, male	2.339 (1.565–3.532)	**< 0.0001**	2.276 (1.408–3.735)	**0.0009**
BMI	1.018 (0.9895–1.047)	0.2192		
CCI	0.9976 (0.9927–1.002)	0.3351		
Chronic steroid therapy	3.059 (1.539–6.223)	**0.0015**	3.402 (1.432–8.459)	**0.0064**
Previous abdominal surgery, yes	1.643 (1.007–2.754)	**0.0467**	1.440 (0.8019–2.654)	0.2304
CRP preoperative	1.003 (1.001–1.005)	**0.0145**	1.001 (0.9975–1.004)	0.7109
Albumin	0.9661 (0.9396–0.9922)	**0.0107**	0.9815 (0.9487–1.014)	0.2733
Creatinine	1.057 (0.9101–1.226)	0.4537		
Duration of surgery	1.004 (1.001–1.006)	**0.0048**	1.002 (0.9985–1.005)	0.3175
Postoperative SOFA score	1.121 (1.050–1.198)	**0.0006**	1.124 (1.039–1.216)	**0.0037**
CRP POD 2	1.005 (1.002–1.007)	**< 0.0001**	1.003 (1.000–1.006)	**0.0480**

Bold text values represent p values less than 0.05

Abbreviations: *OR* odds ratio, *CI* confidence interval, *BMI* body mass index, *CCI* Charlson comorbidity index, *CRP* C-reactive protein, *SOFA* sequential organ failure assessment, *POD* postoperative day

4. **Sex-stratified comparison of postoperative CRP and SOFA scores between patients with or without POI**.

**Fig. 2:**
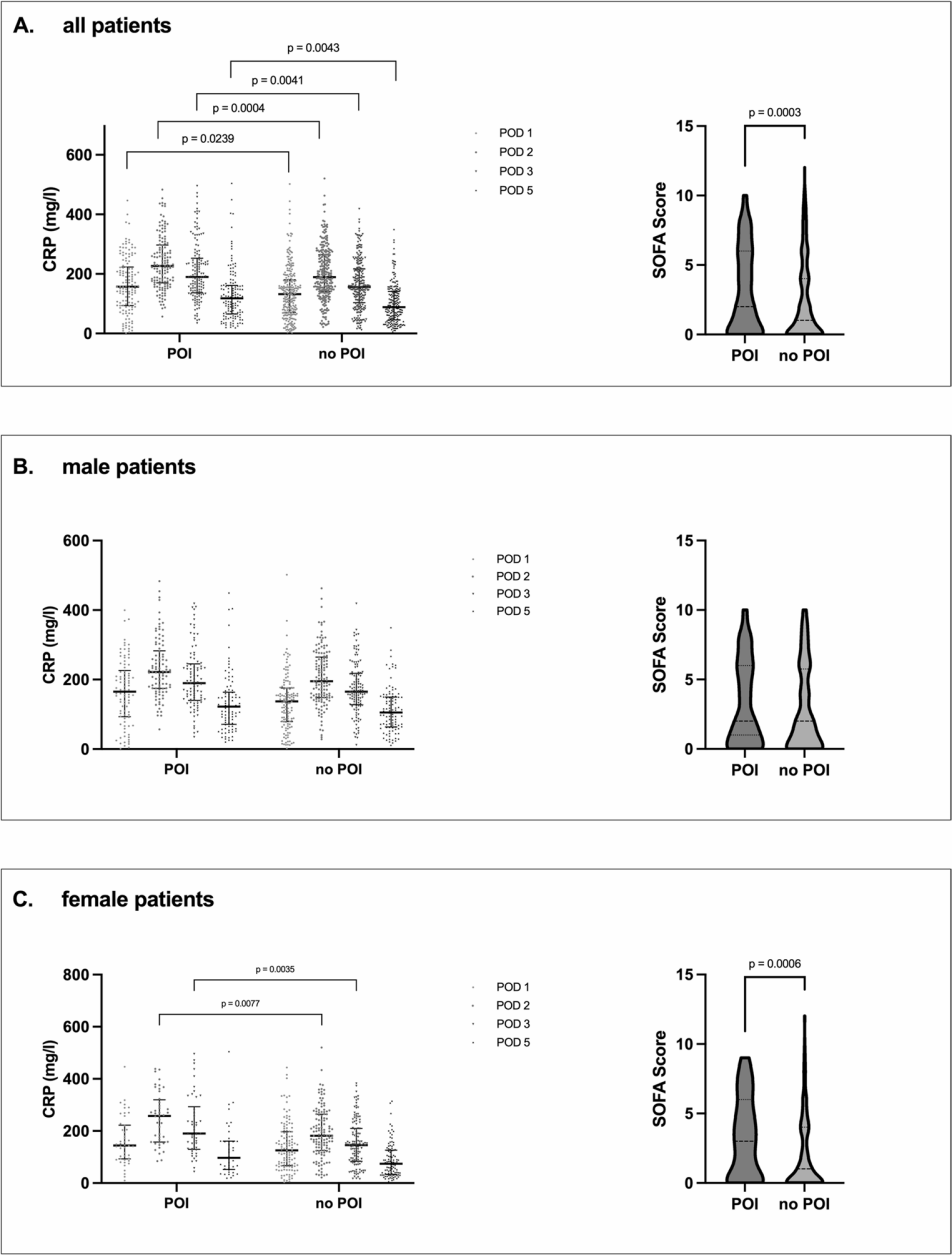
Sex-stratified comparison of postoperative inflammation and organ function between patients with or without POI. C-reactive protein (CRP) levels on postoperative days (POD) 1, 2, 3 and 5 as well as the postoperative sequential organ failure assessment (SOFA) scores were compared in **a** all patients, **b** male patients and **c** female patients with or without postoperative ileus (POI) following emergency surgery for acute bowel obstruction

As male sex seemed to be an independent risk factor for POI in the multivariate analysis, we investigated the differences in bowel recovery between male and female patients concerning postoperative CRP and SOFA scores as further independent POI predictors. In male patients, there was no difference in postoperative CRP or SOFA scores between the subgroups with delayed or timely recovery of bowel function. However, female patients developing POI had significantly higher CRP levels on POD 2 and 3 as well as significantly impaired postoperative organ function (Fig. 2).

5. **Perioperative factors associated with POI depending on the intraoperative procedure.**

We further analyzed whether the previously identified perioperative factors are predictive of POI regardless of whether bowel resection or solely adhesiolysis, detorsion or reposition were performed during emergency surgery for ABO. The complete panel of perioperative parameters included in the univariate analysis in both procedural situations are found in Supplementary Material, Table 2. Table 4 depicts only the parameters significantly associated with POI in univariate analysis. When no bowel resection was needed for the surgical repair of bowel obstruction, 7 perioperative factors including male sex and elevated baseline as well as postoperative CRP levels were significantly associated with POI. However, only chronic steroid therapy seemed to be an independent POI predictor. After bowel resection, male sex, higher postoperative SOFA score and higher CRP levels on POD 2 seemed to independently predict POI (Table 4).

**Table 4:** Univariate and multivariate logistic regression analysis of perioperative risk factors for postoperative ileus following emergency surgery for acute bowel obstruction, with or without bowel resection

Risk factor	no bowel resection (*n* = 243)				bowel resection (*n* = 223)			
	Univariate		Multivariate		Univariate		Multivariate	
	OR (95% CI)	p	OR (95% CI)	p	OR (95% CI)	p	OR (95% CI)	p
Sex, male	2.303 (1.338–4.038)	**0.0025**	1.902 (0.9739–3.801)	0.0632	2.343 (1.292–4.354)	**0.0048**	2.133 (1.064–4.397)	**0.0355**
CCI, %	0.9934 (0.9868–0.999)	**0.0468**	0.9981 (0.9892–1.007)	0.6776				
Chronic steroid therapy	4.997 (1.812–16.05)	**0.0016**	4.783 (1.292–23.17)	**0.0283**				
CRP preoperative	1.004 (1.001–1.008)	**0.0103**	1.001 (0.9964–1.006)	0.6411				
Albumin	0.9476 (0.9108–0.9840)	**0.0049**	0.9776 (0.9292–1,027	0.3739				
Duration of surgery	1.007 (1.002–1.012)	**0.0051**	1.005 (0.9989–1.011)	0.1114	1.004 (1.001–1.008)	**0.0097**	1.003 (0.9996–1.007)	0.0875
postoperative SOFA score					1.200 (1.087–1.329)	**0.0003**	1.187 (1.055–1.340)	**0.0047**
CRP POD 2	1.006 (1.003–1.010)	**0.0007**	1.003 (0.9983–1.008)	0.2106	1.006 (1.003–1.010)	**0.0008**	1.004 (1.000–1.009)	**0.0351**

Bold text values represent p values less than 0.05

Abbreviations: *OR* odds ratio, *CI* confidence interval, *CCI* Charlson comorbidity index, *SOFA* sequential organ failure assessment, *CRP* C-reactive protein

6. **The occurrence of POI correlates with increased postoperative morbidity regardless of the intraoperative procedure**.

**Fig. 3:**
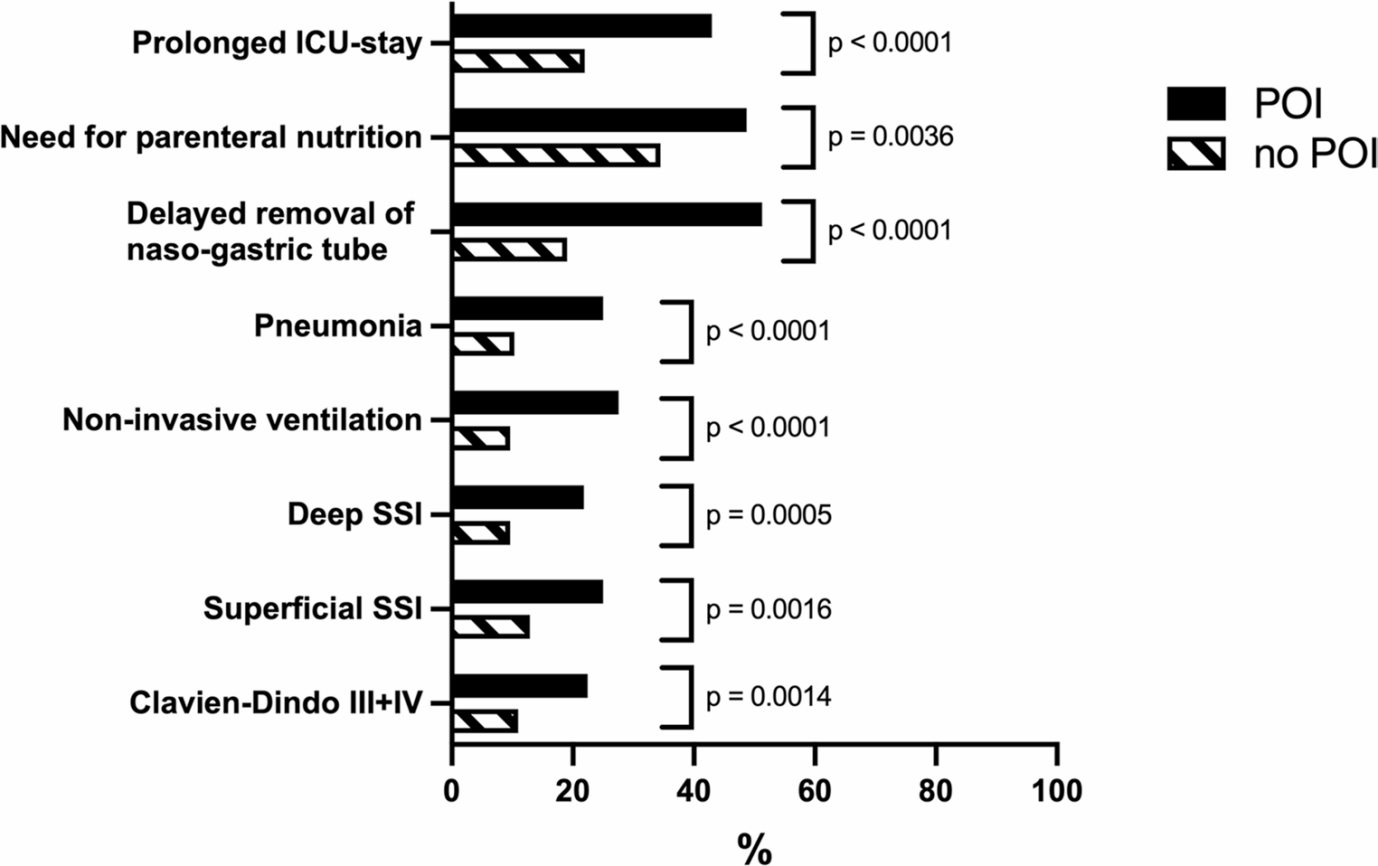
Postoperative morbidity of patients undergoing emergency surgery for acute bowel obstruction. Depicted is the percentage of patients with or without postoperative ileus (POI) developing one of the listed postoperative complications. ICU, intensive care unit; SSI, surgical site infection

Regardless of whether bowel resection was necessary during emergency surgery for ABO, the occurrence of POI positively correlated with a prolonged ICU stay, need of intravenous caloric supplementation and delayed NGT removal (Table 5). The rate of postoperative respiratory complications such as pneumonia and the need for non-invasive ventilatory support were significantly higher in patients with POI (Fig. 3). In terms of surgical complications, correlations were observed between an impaired bowel function and severe surgical complications (Clavien-Dindo^27^ Grade III and IV), including revision surgery in patients undergoing surgical procedures not involving bowel resection. Patients with bowel resection showed a positive correlation with deep and superficial surgical site infections (SSI). There was no correlation between POI and neither in-hospital mortality nor failure to release the patients to their initial home environment (Table 5).

**Table 5:** Correlation analysis of postoperative ileus and medical as well as surgical morbidity following emergency surgery for acute bowel obstruction

		No bowel resection		Bowel resection	
		r	p	r	p
Length of ICU-Stay		0.1914	**0.0027**	0.2805	**< 0.0001**
In-hospital mortality		0.07224	0.2620	−0.0837	0.2131
Release to initial home environment		−0.1180	0.0663	−0.05564	0.4083
Need for parenteral nutrition		0.1491	**0.0203**	0.1571	**0.0197**
Delayed NGT removal (> POD 3)		0.4753	**< 0.0001**	0.3015	**0.0002**
Pneumonia		0.1935	**0.0025**	0.2026	**0.0024**
Need for non-invasive ventilation		0.2176	**0.0006**	0.2656	**< 0.0001**
Re-Intubation		0.07052	0.2745	0.1685	**0.0117**
Readmission to ICU		0.1236	0.0544	0.09678	0.1506
SSI	deep	0.1236	0.0544	0.2228	**0.0008**
	superficial	0.1665	**0.0093**	0.1624	**0.0152**
Need for postoperative imaging		0.1735	**0.0067**	0.2666	**< 0.0001**
Need for revision surgery		0.2035	**0.0014**	0.08299	0.2171
Clavien-Dindo III and IV		0.2032	**0.0015**	0.1313	0.0502

Bold text values represent p values less than 0.05. Surgical complications were reported using the Clavien-Dindo classification system^26^.

Abbreviations: *r* Spearman’s correlation coefficient, *ICU* intensive care unit, *NGT* naso-gastric tube, *POD* postoperative day, *SSI* surgical site infection

## Discussions

The functional recovery of the surgically deobstructed intestine is influenced by patient-, disease-, and procedure-related factors. Identifying these factors and understanding how they affect bowel motility is a necessary step in customizing perioperative treatment to minimize POI-related complications.

Our study is one of the few approaching the issue of POI following emergency surgery and includes 466 patients with ABO that received surgical emergency treatment. Previous studies on similar patient collectives are mainly concerned with the implementation of Enhanced Recovery After Surgery (ERAS^®^) programs in the emergency setting, having POI as a secondary outcome measure [28, 29].

Lohsiriwat et al.’s systematic review and meta-analysis pooled data from three studies involving 818 retrospectively evaluated patients who underwent emergency resection for obstructive colon cancer [28]. Although the meta-analysis indicates a lower POI rate after implementation of ERAS measures, the definition of POI between the included studies was heterogeneous. Only one of them provided a clear definition [30], while the others assessed parameters such as time to first flatus, time to first defecation, time to resumption of normal diet [31] or did not provide a POI definition at all [32]. In lack of a consistent consensus definition for POI [23], we defined POI as the need for intravenous pharmacological stimulation of bowel activity following an insufficient response to initial conservative measures [24].

The first part of our analysis aimed at identifying perioperative parameters associated with an increased risk of bowel dysfunction following emergency surgery for ABO. Multivariate analysis identified male sex, chronic steroid therapy, a high postoperative SOFA score, and elevated CRP levels as potential independent predictors for POI. Interestingly male sex has also been suggested as a risk factor for POI in a meta-analysis of 29,736 patients with elective colorectal resection, along with cardiac comorbidities, previous abdominal surgery, laparotomy, and ostomy creation [33]. While increased surgical complexity due to the narrower male pelvis may explain these findings in elective rectal procedures, this rationale does not apply to our heterogeneous patient group. Although our study cannot establish causality, we observed sex-specific trends: women with POI showed significantly higher postoperative CRP levels and SOFA scores, whereas this was not the case for men.

Inflammation is a central mechanism in POI pathophysiology, largely driven by activation of intestinal macrophages near the myenteric plexus [5, 6]. There is growing evidence of sex differences in innate immune responses, including macrophage behavior after surgical trauma and pathogen invasion. These differences stem from sex chromosome-linked gene expression and the presence of sex steroid receptors in immune cells [34–37]. For example, Toll-like receptor 4 (TLR4), involved in sensing microbial danger signals, is expressed at higher levels in macrophages from males upon lipopolysaccharide (LPS) exposure [38], leading to sex-dependent cytokine production patterns [37–39]. Furthermore, there is increasing evidence that sex hormones influence the composition of gut microbiota as well as the integrity of the intestinal epithelial barrier, leading to lowered microbial virulence and increased resistance to intestinal injury in females [40–42]. The manner and degree in which these mechanisms contribute to the lower incidence of POI in female patients remains an interesting topic for future research.

We identified chronic glucocorticoid treatment, that is frequently used to dampen inflammation, and increased postoperative CRP levels as potential independent risk factors for POI. This strengthens the suggestion that inflammation, likely both systemic and intestinal, plays a significant pathogenic role in POI. It may even have a greater impact in this context than in elective procedures.

In a study on 383 patients who underwent elective colorectal resections, Fujii et al. identified the duration of operation, surgical blood loss, and postoperative serum CRP as factors significantly associated with POI in univariate analysis. However, these factors lost their significance on multivariate analysis [43]. Boersema et al. reported higher levels of postoperative interleukin (IL)−6, leukocyte numbers, and CRP levels in patients with POI after oncologic colorectal resections, though not reaching statistical significance [44].

In our cohort of emergency surgery patients, baseline and postoperative CRP levels were significantly associated with POI in univariate analysis among those not undergoing bowel resection. However, these associations were not confirmed in multivariate analysis. In contrast, among patients requiring resection, elevated CRP on POD 2 emerged as a potential independent predictor of POI. We hypothesize that the inflammatory response in these cases is amplified by bacterial translocation through ischemic bowel segments and by peritoneal contamination from spillage of bowel contents—both of which may further delay functional recovery. However, fecal contamination was documented only in a few isolated cases—4 patients in the POI group and 8 in the non-POI group—and this difference did not reach statistical significance. Bacterial translocation was not systematically assessed because intra-abdominal swabs were not routinely collected.

Due to the very low number of laparoscopically resolved ABO cases, our study does not allow for meaningful conclusions regarding the potential benefits of a laparoscopic approach on postoperative bowel recovery. The overall low rate of laparoscopy in our cohort can be partly explained by the high prevalence of previous abdominal surgeries in both groups, which prompted a primary open approach based on the suspicion of extensive interenteric adhesions.

Whether reduced bowel manipulation during laparoscopy translates into decreased intra-abdominal inflammation and subsequently improved postoperative bowel recovery remains an open question. This hypothesis should be addressed in well-designed randomized controlled trials (RCTs) that also account for the extent of intra-abdominal adhesions. The LASSO trial remains the only RCT to date specifically addressing this topic. However, it predominantly enrolled patients with suspected single-band adhesions [45]. Nonetheless, ongoing efforts by experienced emergency surgeons to expand the indications for a laparoscopic-first approach have been documented [46].

Another important finding of our analysis is the correlation between POI and a series of negative postoperative outcomes observed regardless of whether bowel resection was necessary or not. POI positively correlated with longer ICU stays and the need of parenteral nutritional support. There was also a positive correlation with respiratory complications which can be easily explained through the increased risk of aspiration and impaired respiratory mechanics caused by abdominal distention in patients with POI. Surprisingly the correlation between POI and surgical complications, including those requiring revision surgery was more pronounced in patients not requiring bowel resection. This might be due to the fact that prolonged, extensive adhesiolysis often leads to unnoticed or insufficiently repaired full- or partial-thickness intestinal wall damage as a source of major morbidity [19]. There was no association between POI and in-hospital mortality. While also observing no increase in mortality due to POI alone, Tevis et al. reported a relevant increase in mortality due to the synergistic effect of POI and other postoperative complications [9]. Not to be neglected are the socio-economic implications of POI with a recent meta-analysis reporting an increase of about 60% of total hospital costs due to POI, amounting to a significant global financial burden [10].

While our study is limited by its retrospective design, it offers valuable insights into a relatively underexplored area: bowel function recovery after emergency surgery. Like many other studies, ours is constrained by the absence of a standardized definition for POI. We were also unable to account for all potentially relevant perioperative variables. Future research should consider incorporating additional pre- and postoperative inflammatory markers, quantifying prestenotic bowel distension, and evaluating bacterial translocation as a potential indicator of intestinal barrier dysfunction.

A comprehensive exploration of postoperative bowel recovery, including its complex immunological, genetic, and microbiological underpinnings, is beyond the scope of our study. However, a key insight from comparing our findings with existing literature is that bowel function recovery following emergency surgery differs from that seen in elective procedures. It involves a distinct set of risk factors and complications. This underscores the need for further research focused specifically on POI in emergency surgery patients, with the goal of developing targeted prevention and treatment strategies tailored to their unique risk profiles.

## Conclusion

Male patients, patients under chronic steroid therapy and those with enhanced postoperative inflammation and organ dysfunction are at higher risk of bowel paralysis after emergency surgical treatment of bowel obstruction. POI occurring in these patients is associated with a higher incidence of respiratory and surgical complications. A better understanding of the specifics of bowel function impairment primarily induced by the bowel obstruction itself and seconded by surgical trauma allows for a more targeted implementation of prevention and treatment measures. Our findings provide clear-cut hypotheses for prospective studies that are urgently needed to develop guidelines improving the postoperative outcome of emergency surgery for ABO.

## Supplementary Information

Below is the link to the electronic supplementary material.

ESM 1 (DOCX 16.7 KB)

## Data Availability

The raw datasets generated and analyzed during the current study are available from the corresponding author upon reasonable request, without undue reservation.
