# Supplementary material for: Postoperative ileus after emergency surgery for acute bowel obstruction: a case-control study of predictors and impact on recovery
Source: Langenbecks Arch Surg. 2025 Sep 1;410(1):260. doi: 10.1007/s00423-025-03851-0 (PMC12401765; doi:10.1007/s00423-025-03851-0)
Supplement: Supplementary file 1 — (DOCX 16.7 KB) [file 423_2025_3851_MOESM1_ESM.docx]

**Supplementary Material, Table 1**

**Univariate logistic regression analyzing the association between laboratory parameters measured on PODs 1, 2, and 3 and postoperative ileus (POI) following emergency surgery for bowel obstruction**

| Risk factor | Univariate | |
| --- | --- | --- |
|  | OR (95% CI) | p |
| POD 1 |  |  |
| Leukocyte count | 1.007 (0.9785 - 1.036) | 0.6216 |
| CRP | 1.003 (1.001 - 1.006) | **0.0052** |
| POD 2 |  |  |
| Leukocyte count | 1.019 (0.9853 - 1.055) | 0.2668 |
| CRP | 1.005 (1.002 - 1.007) | **<0.0001** |
| Albumin | 0.9902 (0.9458 - 1.036) | 0.6698 |
| Creatinine | 1.063 (0.9202 - 1.230) | 0.3975 |
| Bilirubin | 1.439 (0.9548 - 2.187) | 0.0818 |
| POD 3 |  |  |
| Leukocyte count | 1.035 (0.9955 - 1.077) | 0.0833 |
| CRP | 1.003 (1.001 - 1.006) | **0.0013** |

Bold text values represent p values less than 0.05.

Abbreviations: OR, odds ratio; CI, confidence interval; POD, postoperative day; CRP, C-reactive protein.

**Supplementary Material, Table 2**

**Univariate logistic regression analyzing the impact of perioperative variables on postoperative ileus (POI) after emergency surgery with or without bowel obstruction.**

| Risk factor | no bowel resection (n = 243) | | bowel resection (n = 223) | |
| --- | --- | --- | --- | --- |
|  | OR (95% CI) | p | OR (95% CI) | p |
| Age, years | 1.009 (0.9941 - 1.025) | 0.2318 | 1.010 (0.9894 – 1.031) | 0.3555 |
| Sex, male | 2.303 (1.338 - 4.038) | **0.0025** | 2.343 (1.292 – 4.354) | **0.0048** |
| BMI | 0.9988 (0.9592 - 1.039) | 0.9518 | 1.038 (0.9972 - 1.083) | 0.0680 |
| CCI | 0.9934 (0.9868 - 0.9999) | **0.0468** | 1.001 (0.9939 - 1.009) | 0.7008 |
| Chronic steroid therapy | 4.997 (1.812 - 16.05) | **0.0016** | 2.028 (0.7406 - 5.395) | 0.1640 |
| Previous abdominal surgery | 1.680 (0.8138 - 3.689) | 0.1641 | 1.492 (0.7673 - 3.039) | 0.2429 |
| CRP preoperative | 1.004 (1.001 - 1.008) | **0.0103** | 1.002 (0.9987 - 1.005) | 0.2363 |
| Albumin | 0.9476 (0.9108 - 0.9840) | **0.0049** | 0.9773 (0.9377 - 1.015) | 0.2436 |
| Creatinine | 1.052 (0.8220 – 1.341) | 0.6700 | 1.075 (0.8827 - 1.311) | 0.4474 |
| Duration of surgery | 1.007 (1.002 - 1.012) | **0.0051** | 1.004 (1.001 - 1.008) | **0.0097** |
| Postoperative SOFA score | 1.069 (0.9775 - 1.168) | 0.1434 | 1.200 (1.087 - 1.329) | **0.0003** |
| CRP POD 2 | 1.006 (1.003 – 1.010) | **0.0007** | 1.006 (1.003 - 1.010) | **0.0008** |

Bold text values represent p values less than 0.05.

Abbreviations: OR, odds ratio; CI, confidence interval; BMI, body mass index; Charlson comorbidity index; CRP, C-reactive protein; SOFA, sequential organ failure assessment; POD, postoperative day.
